# P-201. Development of an Interactive Application to Visualize the Geographic Location of *Clostridioides difficile (C.diff)* Cases at a Large Academic Hospital

**DOI:** 10.1093/ofid/ofae631.405

**Published:** 2025-01-29

**Authors:** Courtney Hebert, Priti Singh, Justin Smyer, Jennifer Martin, James B Odei, Megan E Gregory, Michael F Rayo, David Kline

**Affiliations:** The Ohio State University, Columbus, Ohio; The Ohio State University, Columbus, Ohio; The Ohio State University Wexner Medical Center, Columbus, Ohio; The Ohio State University Wexner Medical Center, Columbus, Ohio; The Ohio State Univeristy, Columbus, Ohio; University of Florida, Gainesville, Florida; The Ohio State University, Columbus, Ohio; Wake Forest University School of Medicine, Winston-Salem, North Carolina

## Abstract

**Background:**

Despite the growth of digital healthcare data, surveillance for healthcare associated infections (HAIs) often consists of reviewing cases using lists, charts, or tables. For infections, such as *C.diff* which can be transmitted patient-to-environment and patient-to-patient, it can be useful to visualize the location of these infections to detect spatial clustering. Here we discuss the development of a web application (GeoHAI) for infection preventionists (IPs) to visualize C.diff in rooms over time and quantify the burden of *C.diff* in these rooms.

**Figure 1:**
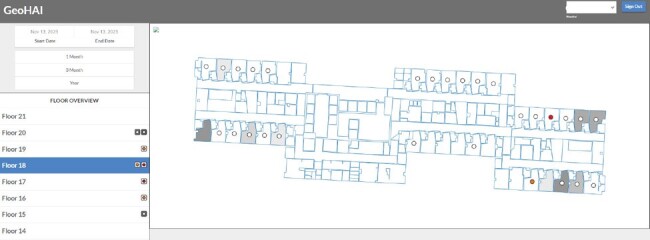
Screenshot of GeoHAI application (Development environment with simulated case data)

This screenshot shows a view of a floor within one of the hospital buildings. Each circle in a room relates to a patient who was in that room on the chosen date. Clicking on a circle will give details on that patient (Patient ID, admit/discharge date, room number, date room was entered and exited, as well as whether the patient had C. diff (HO- or CO-CDI) and what was the date of the positive test). Orange circles indicate CO-CDI, red are HO-CDI. Shading corresponds to the cumulative number of hours patients with C. diff (HO- or CO-CDI) were in each room for the previous 30 days. The user can also look at the shading for 90 days or 365 days by choosing one of those options in the upper left-hand corner. The shading is intended to give a sense of overall burden of C. diff in each room. The list of floors on the left panel allows the user to switch between floors, and boxes in this area give a preview of high interest rooms on that floor. The building can be selected from the drop-down box in the upper right corner.

**Methods:**

Development of GeoHAI consisted of (1) processing clinical and geographic data; (2) software development; and (3) deployment within the medical center (MC).

*(1)* Floor plan data was converted to GeoJSON files which contain coordinates to draw polygons corresponding to rooms within the hospital. Rooms were given a spaceID, a unique number for each building-floor-room. Clinical and patient location data include patient ID, admission/discharge date, *C.diff* result, and entry/exit times for room transfers. We wrote R code to clean the data and identify hospital onset vs community onset *C.diff* (HO-CDI, CO-CDI), to link patient room to spaceID, and to calculate the historical burden of C.diff in each room, defined as the cumulative number of hours that patients with active *C.diff* spent in each room. (2) With IP input, we created design specifications which were used to develop the application. Node-RED was used to load clean, clinical data from CSV files into the application database. *(3)* IT worked with software developers to host the application on the MC’s cloud servers using Azure DevOps.

**Results:**

GeoHAI is accessed by authorized users in development and production environments on the MC network. Users choose from 42 unique floors in 6 buildings, can select a patient within a room to see if they had *C.diff* during their stay, the date of C.diff, and room entry/exit date. The degree of room shading correlates to the cumulative hours patients with *C.diff* were in a room (Figure 1). New data can be loaded via an intuitive web interface.

**Conclusion:**

GeoHAI displays *C.diff* cases in a large academic hospital. We next plan to study how GeoHAI impacts HAI investigation, to expand GeoHAI features, and to add additional HAIs.

**Disclosures:**

**All Authors**: No reported disclosures

